# A case study: what is leached from mallee biochars as a function of pH?

**DOI:** 10.1007/s10661-018-6681-8

**Published:** 2018-04-18

**Authors:** Caroline Lievens, Daniel Mourant, Xun Hu, Yi Wang, Liping Wu, Angelina Rossiter, Richard Gunawan, Min He, Chun-Zhu Li

**Affiliations:** Fuel and Energy Technology Institute, Curtin University, GPO Box U1987, Perth, WA 6845 Australia

**Keywords:** Biochar, Leaching, pH, Macronutrients, ICP-OES, GC-MS, UV-fluorescence, Mallee

## Abstract

Biochar is widely considered as a soil amendment. This study aims to investigate the leaching of macronutrients (K, Mg and Ca) and organics from biochars produced from mallee biomass (wood, leaf, bark) in a fluidised-bed pyrolyser at 500 °C. Biochars were soaked in solutions of varying pH values and shaken for a pre-set period of time ranging from 1 h to 4 weeks. The initial pH values of the leaching solutions used (3.4, 5.5, 7 and 8.5) covered the pH range of the soils in the Wheatbelt region of Western Australia (WA). For these bark, leaf and wood biochars, we can conclude that the biochars have a liming capacity for the acid soils of the WA Wheatbelt, depending on the feedstock. The maximum leachabilities and leaching kinetics of the macronutrients K, Mg and Ca depend on the pH of the solution in which biochar was soaked. Apparently, Ca, K and Mg in biomass are converted into different species upon pyrolysis, and the biomass species are critical for the extent of the leachability of macronutrients. Further, the chemical form of each nutrient retained in the biochars will dictate the kinetics as a function of soil pH. This study’s GC/MS analysis of solvent extraction of the biochars showed potential toxicity due to the leaching of light organic compounds when biochars are added to soils. Furthermore, this study also showed the influence of pH on the leaching of large aromatic organics from the biochars. Apart from the pH of leaching solution, the influence of the biomass feedstock on the leaching kinetics of large aromatic organics from biochars was demonstrated. These leached aromatic organics were characterised by UV-fluorescence spectroscopy.

## Introduction

The application of biochars as an amendment for poor soils is receiving an increased consideration worldwide, at least partly due to the pressures to reduce the emission of greenhouse gases. The potential of biochar for soil conditioning, such as reducing soil acidity, improving soil cation exchange capacity, soil pH, soil nutrient retention, water holding capacity and soil microbial habitat, makes biochar application an interesting option for increasing soil productivity and profitability (Abdel-Fattah et al. 2015; Lehmann 2007; Lehmann and Joseph 2009; Lehmann et al. 2011; Uchimiya et al. 2010, 2011).

The pyrolysis of different feedstocks (Lievens et al. 2008, 2015) under widely different conditions, such as temperature (Hagner et al. 2016; Lievens et al. 2009), would result in a large variety of biochars, all with specific characteristics in regard to inorganic compositions. Some biochars are known to sorb organic (Buss et al. 2016; Mohan et al. 2014) and inorganic molecules (Silber et al. 2010; Velghe et al. 2012), retaining nutrients against leaching losses. These are all important factors in improving crop productivity and soil fertility. The release of nutrients from biochar, especially inorganic nutrients, would depend on the pH of moisture (water) in the soil (Randolph et al. 2017). The department of Agriculture and Food of Western Australia (WA) found that the pH of topsoils in the WA Wheatbelt ranges between 3.8 and 8.1, with 80% of the topsoils falling below the critical surface pH_CaCl2_ of 5.5 (Gazey and Andrew 2009). Little is known about the effects of biochar addition to the soils, including potential toxic effects to the soil, as a function of the soil pH.

Previous studies have demonstrated that organics can be leached from biochars when in contact with water (Hartley et al. 2016; Lievens et al. 2015). The addition of biochar to soil could have potential toxic effects due to the leaching of organics. For example, during the biomass pyrolysis for biochar production, some of the organic-based tars may condense on the biochars’ surface, making it vital to understand the leachability of organic matter in biochars when they come in contact with water. Soils can have different pH and the leaching of organics could be pH dependent. The identification and characterisation of organic matter leached out should also be understood as such information is essential in assessing the toxicity of the leached-out organic matter to the soils and its biota. Little to no such data exists; no experiments have been performed to investigate the leachability of organic compounds, as a function of soil pH, from biochars to our knowledge. Pyroligneous acid (pyrolysis liquids) has been added to soils, used to control pests and diseases (Focht 1999). It has been illustrated that when bio-oils are added to soils, that these pyrolysis compounds are easily metabolised by soil microbes and bacteria, and even intensify the respiratory metabolism of the soils (Fischer and Bienkowski 1998; Steiner et al. 2008). However, some of the typical biomass pyrolysis liquid compounds, e.g. phenols, have been on the list of priority pollutants for a long time, due to their potential to cause many adverse effects to human health and the environment (Djokic et al. 2013). Concentrations as low as 0.005 mg l^−1^ are causing adverse effects to aquatic environment, while 0.8 mg kg of soil^−1^ is considered toxic (Djokic et al. 2013).

Mallee was used in this study since mallee eucalypt is a small, multi-stemmed tree that is currently grown on marginal land, but also grown in the Wheatbelt of WA as a way to control soil salinity and offer protection to crops. It can be harvested every few years and the tree resprouts from the cut stump; in essence, mallee wood is in abundance, hence much biochar can be produced locally. A distinction between wood, leaf and bark needs to be made since they have different anatomical features (Lievens et al. 2011), making the distribution of macronutrients different depending on the plant part. This is because some of these elements prefer to reside in water-rich tissues or are central to chlorophyll (Ghisalberti 1996; Kramer and Kozlowski 1979). The hypothesis is that pyrolysis of different plant parts will result in different biochars with different forms of macronutrients which will behave differently when soil solution pH is altered.

In this study, we investigated the potential toxicity due to light bio-oil compounds leaching from bark, leaf and wood biochars, in which the biochars were solvent washed after which the leachates were GC/MS analysed for light bio-oil compounds. Further, the leaching of macronutrients (Ca, K, Mg) and aromatic organics from the respective biochars as a function of the pH of the leaching solution and time was investigated. To exclude all other soil parameters, except pH, a simple set-up was devised in which biochars were stirred in certain pH solutions. Four pH buffer solutions were prepared: 3.4, 5.5, 7 and 8.5, covering the whole range of soil pH of the WA Wheatbelt. The influence of pH was investigated in regards of the nature, concentration and kinetics of leached inorganic compounds from biochars originating from the three feedstocks. The released aromatic organics were characterised by UV-fluorescence spectroscopy.

## Experimental section

### Preparation of biochars

Biochars were produced from the pyrolysis of mallee bark, leaf and wood in a fluidised-bed pyrolyser. The bark, leaf and wood biochars (*Eucalyptus loxophleba ssp. gratiae*, 180–600 μm particle size) were produced at 500 °C. The residence time of the solids of biomass/biochar during pyrolysis was both 1.4 s in the reactor for 500 °C. The detailed description of the pyrolyser and the various experiments can be found elsewhere (Garcia-Perez et al. 2008; He et al. 2012; Mourant et al. 2013).

### Leaching experiments

Biochars were immersed and shaken in solutions (biochar/solution ratio of 1:20 by weight) of pH 3.4, 5.5, 7 and 8.5. After the pre-set soaking time (1 h, 1 day, 1 week, 2 weeks or 4 weeks) was elapsed, the solutions were filtered through a 0.2-μm Supor® membrane filter. After filtration, the biochar wash solutions were kept in a refrigerator (4 °C). The various buffers, with their buffering capacity, used to maintain the required solution pH are described in Table 1. All leaching experiments were performed in duplicate.

**Table 1:** Buffer solutions used for the leaching of biochars at different pH levels

Determined pH	pH buffer solutions	Buffering capacity
3.4	500 mL 0.2 M glycine–50 mL 0.2 M HCl, diluted to 1 L	2.2–3.6
5.5	95 mL 0.1 M acetic acid–905 mL 0.1 M sodium acetate	3.7–5.6
7	500 mL 0.1 M NaH_2_PO_4_–300 mL 0.1 M NaOH	5.8–8.0
8.5	500 mL 0.2 M glycine–80 mL 0.1 M NaOH, diluted up to 1 L	8.4–10.6

### Characterisation of biochars: proximate and ultimate analyses

The ash yield (CEN 14775) and moisture content (CEN 14774) of biochars were determined by an external laboratory according to the European standards. Carbon, hydrogen and nitrogen were determined using a Leco Truspec Analyser.

The total elemental concentrations of K, Ca, Mg in biochars were determined after the acid digestion of the biochars. For the acid digestion, 50 mg of biochars was weighed in small Pyrex beakers covered with Pyrex watch glasses. To each biochar sample, 2 mL of HNO_3_ (69%, Suprapur, Merck) was added and was then heated to a temperature of approximately 150–175 °C in a fume cupboard. After the nitrous gases had dissipated, the beakers were rinsed with ultrapure water (Millipore water; 18.2 MΩ.cm). After the evaporation of water, more nitric acid was added and the procedure was repeated, during which the biochars became more and more digested and the digestion solutions changed colour from brown, brownish red, dark red, orange to yellow. For the more resilient biochars, where the acid digestion solutions remained dark in colour even after HNO_3_ had been added repeatedly, HClO_4_ (70%, redistilled, Aldrich) was added. HClO_4_ was always added as a mixture: HNO_3_-HClO_4_ (4:1) (Bock 1979). After the digestion was completed, the dry yellowish residues in the beakers were dissolved in ultrapure water. Following this process, the solutions were analysed with ICP/OES. All digestions were performed in duplicate, and the results were corrected with blank experiments.

### GC/MS analysis of leachate from leaching biochars with organic solvent

The biochars were washed with methanol (LC Chromasolv, HPLC quality of Merck) or methanol/chloroform (HPLC quality of Merck) for 72 h (biochar/solution ratio of 15:100). After the stirring time had lapsed, the solutions were filtered over a 0.2-μm Supor® membrane filter. The analysis of the compounds in the biochar leaching solutions was carried out using an Agilent GC-MS (6890 series GC with 5973 series MS detector), with a 30 m × 0.25 mm i.d HP-Innowax capillary column (0.25 μm cross-linked polyethylene glycol). The analysis consisted of injecting 1 μL of sample under the following conditions: split-less, initial oven temperature of 40 °C with a holding period of 3 min, then heated at a heating rate of 10 °C min^−1^ to 260 °C and held for 5 min. A solvent delay of 3.6 min was employed. Masses were scanned from 15 to 500 mass units. The identification of each compound was achieved by the match of its mass spectrum with that in the spectral library and was further confirmed by injecting the standard when available. Standard solutions were used to obtain the calibration curves to calculate the concentrations of the compounds of interest. It was difficult to obtain standards for all the compounds identified in the GC-MS chromatograms. In those cases, the signal intensity (peak area) of the compound was used as a measure of the changes in their concentration as a function of reaction conditions.

### Analysis of leachates from the leaching of biochars

#### pH

The pH of the buffer solutions and biochar leachates was measured using a TPS smart Chem-Lab Laboratory analyser with a combination pH sensor.

#### ICP/OES

The nutrients (Ca, Mg, K) in the biochars (after digestion) and those leached from the biochars were determined with a Perkin Elmer Optima 7300 DV (Dual View) ICP/OES. Ca, Mg and K were all measured using a normal purge method. Magnesium and calcium were measured axially, potassium radially, to avoid the underestimation of K as a result of recombination of ions and electrons in the plasma. Calibration was performed by diluting a 10,000-mg/L Merck ICP/MS standard solution.

#### Analysis of leachates from the leaching of biochars with UV-fluorescence

In this study, the UV-fluorescence spectra of biochar leaching solutions with an initial pH value of 3.4, 5.5, 7 and pH 8.5 were recorded using a Perkin-Elmer LS50B spectrometer. The obtained biochar wash samples were diluted with ultrapure water to obtain a linear relationship between UV fluorescence intensity and concentration. The synchronous spectra were recorded with a constant energy difference of − 2800 cm^−1^. The slit widths were 2.5 nm and the scan speed was 200 nm min^−1^. The “wavelength” shown for each spectrum refers to that of the excitation monochromator. Wavelength is a brief indication of the aromatic ring systems (Li et al. 1993; Wang et al. 2012) although a clear delineation about size of aromatic ring systems and wavelength ranges is impossible. Intensities shown are averages of duplicate experiments. At the same concentration, the fluorescence intensity was divided by the amount of char to express all fluorescence intensity on the basis of “per gram of char”.

## Results and discussion

### Characteristics of biochars

Table 2 gives the proximate and elemental analyses of the biochars produced in the fluidised-bed pyrolyser. Wood biochar had lower ash yield and oxygen content than other biochars produced under similar conditions. All C/N ratios were above the critical limit of 20, the limit above which the immobilisation of N by microorganisms occurs and nitrogen is no longer available to plants (Lehmann and Joseph 2009). In essence, mallee bark, leaf and wood biochars are a poor choice for soil N-replenishment if used as is.

**Table 2:** Proximate and elemental analyses of biochars used in this study

Feedstock	Bark	Leaf	Wood
Moisture (%)	6.1	6.2	3.75
Ash (% db)	25.1	20.9	15.15
C (% daf)	76.9	81.2	85.9
H (% daf)	2.9	3.2	6.5
N (% daf)	0.3	2.3	0.2
O^a^ (% daf)	20.0	13.5	7.4
C/N	428	43	669
H/C	0.46	0.47	0.49
O/C	0.19	0.12	0.06

^a^Oxygen % (by difference)

Table 3 gives the elemental analyses of the biochars produced in the fluidised-bed pyrolyser, as are the respective yields of bark, leaf and wood biochars. It is very clear from Table 3 that biochar of wood had the lowest concentrations of Ca, Mg and K, which are macronutrients for plants, among all biochars investigated in this study. The loss of macronutrients upon pyrolysis depends on the biomass feedstock. Apparently, Ca, Mg and K in leaf biomass are concentrated in the biochar without apparent loss. Macronutrient Ca in bark and wood, however, is partly lost during pyrolysis, as is potassium after wood pyrolysis.

**Table 3:** Macronutrient concentrations (mg/kg) in the biomass and biochars (produced at 500 °C) prepared in a fluidised-bed pyrolysis reactor and the yields of the biochars

	Bark	500 °C	Leaf	500 °C	Wood	500 °C
K	2176 ± 132	6920 ± 50	3865 ± 310	17,000 ± 130	1095 ± 70	4700 ± 111
Ca	33,905 ± 2650	56,400 ± 90	5949 ± 271	26,300 ± 100	2490 ± 150	13,200 ± 190
Mg	1094 ± 58	3040 ± 40	1194 ± 56	4800 ± 20	355 ± 20	1920 ± 1
Char Yields		33%		27%		15%

Errors shown represent the standard deviations of determinations

### Light organic compounds leached out from biochar using organic solvents

Bark and wood biochars were washed with methanol, leaf biochars with methanol/chloroform, which is known to be a very effective mixture, better than methanol and/or acetone, to dissolve the leaf pyrolysis bio-oils. GC/MS analysis was performed to obtain information about the bio-oil compounds condensed on mallee biochars. These results give information about the light bio-oil fraction that has been condensed on mallee biochars during the fast pyrolysis process. They also indicate the possible toxicity of biochars when added to the soil due to the leaching of these physically adhering bio-oil compounds. No information about possible condensed large multi-fused aromatic structures were obtained since these compounds have very high boiling points, which are not detected in the GC/MS. All major GC/MS-detected compounds in the methanol or methanol/chloroform leachates of bark, leaf and wood biochars have been quantified. Table 4 lists the concentrations of these compounds, expressed as μg/g of biochar or peak area/g of biochar.

**Table 4:** All major GC/MS identified compounds (μg/g char or area/g char) in the solutions after leaching the biochars with solvents

	Bark μg/g char *(methanol leaching)*	Leaf μg/g char *(methanol leaching)*	Wood μg/g char *(methanol leaching)*
Phenol	8.5	16	52
Levoglucosan	58	75	350
Compound	Area/g biochar		
Acetic acid	82,400	206,000	10,880,000
Furfural	n.d.	n.d.	2,604,000
Propanoic acid	n.d.	n.d.	883,000
Glycol	n.d.	n.d.	2,271,000
3-methyl butanoic acid	n.d.	33,000	n.d.
Cylcopentene	n.d.	n.d.	88,700
4-Methoxyphenol	3870	n.d.	n.d.
3,4-Dimethoxyphenol	4120	70,000	1,834,000
1,2,4-Trimethoxybenzene	n.d.	26,000	403,000
OHmethylfurfural	n.d.	n.d.	185,000
4-OH-3-methoxybenzaldehyde	n.d.	16,100	339,000

*n.d.* not detected

The results in Table 4 show that compounds physically adhered to the biochar were very dependent on the feedstock from which they originated. For example, furfural (and derivates) and acetic acid were detected in much higher concentrations in the wood biochar leachate than the bark and leaf leachates. Furfural is formed by dehydration of the xylose unit (hemi-cellulose component) (Demirbas 2005). The formation of acetic acid results from the elimination of acetyl groups originally linked to the xylose unit (Matsuzawa et al. 2001) or after dehydration and decarbonylation of cellulose (Demirbas 2001). Taking a closer look at the chemical build-up of the different mallee plant parts, it is clear that the differences in concentrations in furfural and acetic acid in the biochar leachates can be found from the variation in concentrations of hemi-cellulose/cellulose in the feedstock. In particular, the wood fraction of mallee contains 63% of hemi-cellulose/cellulose, far more than the leaf (30%) and bark (46%) fraction of the same mallee trees (Lievens et al. 2011).

Two possible explanations may be given for detecting bio-oil compounds in the char fraction. The short particle residence time in the fluidized-bed reactor, at the order of seconds, may not be long enough for the pyrolysis reactions to go to completion and/or for the pyrolysis products to be transferred out of the particles. The second possibility is that these compounds listed in Table 4 have their origin from the re-condensation of bio-oil vapours before the bio-oil vapour and biochar were separated.

Comparing the light bio-oil compounds that adhered on the mallee biochars (Table 4), a variety of compounds with higher concentrations adhered to wood biochar surfaces. These data indicate that wood behaved differently from bark and leaf under the same pyrolysis conditions. Having experienced higher weight losses, the wood biochar may be more porous than those of leaves and bark, giving rise to better adsorption characteristics (He et al. 2012; Mourant et al. 2011, 2013). Alternatively, the bio-oil vapours from the wood biochar contained higher concentrations than those species listed in Table 4, giving better chance for them to be physically adhered to char in comparison to bark and leaves biochar.

The phenolic compounds leached out of mallee biochars listed in Table 4 are on the priority list of hazardous substances (Djokic et al. 2013). These compounds could cause environmental concerns when maximum concentrations are reached in soil or groundwater. Concentrations as low as 0.005 mg l^−1^ can cause adverse effects to an aquatic environment, while 0.8 mg kg of soil^−1^ are considered toxic (Djokic et al. 2013). The concentration of phenol leached from bark, leaf and wood biochars with organic solvents range from 8 to 52 mg/kg biochar depending on the biochar species. These data serve to demonstrate further that the actual amounts of light species that are contained in biochar do vary with mallee source feedstock.

The exact possible toxicity of the (light) bio-oil compounds after the biochar has been applied to the soil would depend on many complicated factors. For example, surface adhering pyrolysis condensates, including water soluble compounds (e.g. carbonyls, alcohols and sugars), can easily be metabolised by soil microorganisms, as discussed intensely in literature (Fischer and Bienkowski 1998; Focht 1999; Steiner et al. 2008). Furthermore, the type of biochar or soil characteristics, soil pH and biota, also have an influence on the biodegradability of phenolics (Baker and Mayfield 1980). Fischer and Bienkowski (1998) investigated the toxicity and intensity of soil metabolism when exposed to aromatic hydrocarbons, such as benzenes, phenolics, furan derivates, naphthalene and numerous polycyclic aromatic hydrocarbons. All these compounds can be consumed by strains of prototrophic bacteria occurring in the soils, as these compounds can be used as food by soil organisms. The pyrolysis compounds appear to be a metabolizable substrate for the microbial community. The turnover time of these substrates is likely to be on the order of one to two seasons and will not determine community composition for any length of time (Lehmann and Joseph 2009), unless frequently reapplied.

### Characterisation of leachates of biochars

#### pH of the leachates

**Fig. 1:**
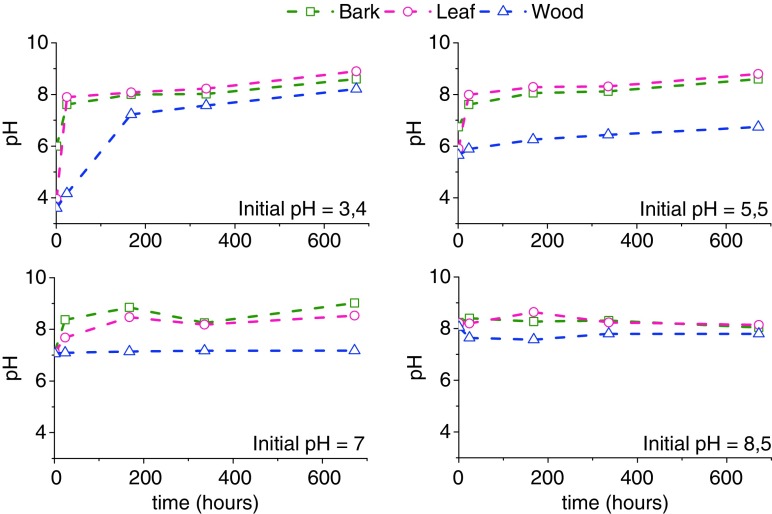
Changes in the pH of leachates as a function of leaching time during the leaching of biochars from the pyrolysis of wood, leaves and bark. The trend lines in all figures have simply connected the datum points and do not represent any model calculations

Figure 1 depicts the evolution of pH value of leachates as a function of leaching time. For the buffer solutions with pH of 3.4, we saw a fast increase in pH reaching a pH of > 7 after 1 week, illustrating the liming capacity of these biochars. In other words, the amounts of K, Mg and Ca leached out from the biochars had far exceeded the buffering capacities of the buffer solutions used at the beginning of the leaching trials. In general, for all biochars, the pH increased when adding biochars to solutions, making the leachates alkaline, with the exception of the pH 5.5 leachate of wood biochar, where the pH remained slightly acidic as a function of time. While the pH of the leachates of other two biochars could go well above 8, the leachates from wood biochar were well below 8 under the experimental conditions investigated. The ability to change the pH of the leachates would be related to the ion-exchange ability of the biochars. However, organic compounds can be adsorbed to the surfaces of biochars (Lievens et al. 2015). As discussed earlier, the biochars adsorbed light organic compounds which could potentially be leached when biochars are added to soils. These leachates showed the leaching of acetic acid (Table 4), increasing the buffering effect of pH solutions. The potential leachable concentration of acetic acid was highest in wood biochar where the pH remained below 8 under the experimental conditions investigated.

These trends illustrate that the liming effects of biochars would change with the biomass feedstock properties. It should be pointed out that the changes in the pH of the leachates are related to both the amounts of macronutrients (K, Mg and Ca) as well as the pyrolysis liquids being leached out. Moreover, the changes in pH of the leachates is most likely linked the speciation of the macronutrients and the chemical forms of the nutrients in the biochars. For example, two H^+^ would be required to ion exchange one divalent species (Ca^2+^ and Mg^2+^) or two monovalent species (K^+^) in the biochar. The exact nature of functional groups in the biochars that bond macronutrients could also affect the pH of the resulting leachates. In order to better understand the leaching process of macronutrients, the overall leachability and the leaching kinetics were investigated. These are important information in terms of recycling of nutrients from biochar applied to the soil.

#### Maximum leachability of elements—an overview of the leaching process

Table 5 shows the maximum amounts of K, Ca, Mg in biochars, which have been leached out, under the conditions investigated, into the leaching solutions at different initial pH values, expressed as % of these elements found in the corresponding biochars before leaching.

**Table 5:** Maximum percentages of K, Ca, Mg in biochars that have been leached at different initial pH values

		pH 3.4	pH 5.5	pH 7	pH 8.5
Bark 500 °C	K (%)	77	91	88	72
	Ca (%)	8	8	1	1
	Mg (%)	18	18	6	9
Leaf 500 °C	K (%)	86	82	89	73
	Ca (%)	5	7	2	1
	Mg (%)	15	13	11	7
Wood 500 °C	K (%)	100	100	100	83
	Ca (%)	20	15	3	4
	Mg (%)	17	14	12	7

The concentrations of these elements leached from biochars, within the leaching time investigated, were highly dependent on the pH of the leaching solution and biomass feedstock to prepare the biochars (Table 5). Potassium leaching characteristics are dominated by the biomass feedstock to prepare the biochars in the leaching solution since for wood biochars, almost maximum leachability is reached compared to leaf and bark biochars, and independent of the pH of the buffer solutions.

For calcium, the leachability is remarkably low in the pH buffer solutions under investigation. Apparently, pyrolysis rendered the bulk of calcium-macronutrient unavailable to the environment in which it is leached. Ca, e.g. the majority could be present as carbonate as it becomes insoluble at pH > 6.8, as these pH values are found for most of the leaching buffer solutions (Fig. 1) in this research. Magnesium leachability seems independent of the biomass of which it originates. The speciation of Mg could not be determined. Speciation could be investigated with SEM-EDX; however, this technique was not available to us during the course of this particular set of experiments.

The change in leachability of macronutrients as a function of pH and biochar type gives a strong indication that these macronutrients are converted into different speciation upon pyrolysis.

These data provide an overview of the leaching process. While the pH value determined the concentration for H^+^ that was available for ion-exchange and the biomass properties determined the chemical forms of the macronutrients in the biochars to be leached. The exact roles of each of these parameters would be better understood by considering the leaching kinetics in the next section.

#### Leaching kinetics of elements

**Fig. 2:**
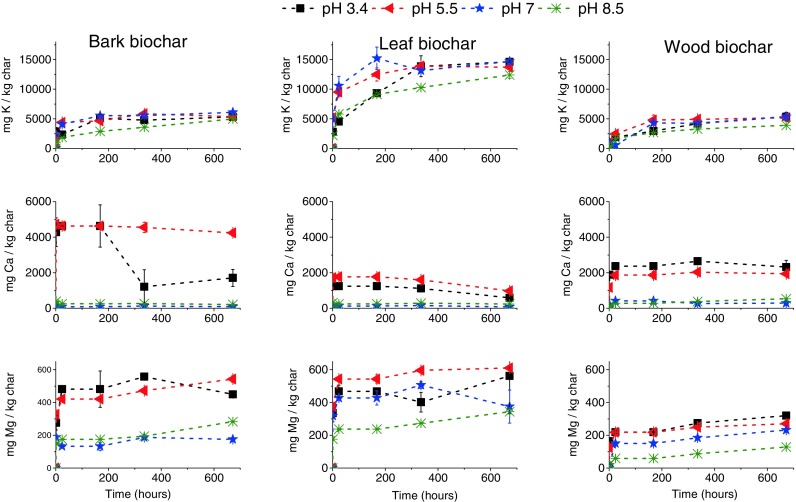
Leaching kinetics of K, Ca, Mg (mg/kg char) from 500 °C biochars at different initial pH values

Figure 2 illustrates the leaching profiles for K, Ca, Mg from bark, leaf and wood (500 °C) biochars. For the leaching of potassium, the total amounts of K leached out from biochar increased gradually with time at all initial pH levels. The (initial) pH value played a role in the leaching rates, however small. It appears that the leaching rate generally decreased with increasing pH value. The leaching in the buffer having an initial pH of 8.5 was the slowest.

Calcium leaching kinetics in the buffer solutions having initial pH values of 7 and 8.5 followed very similar trends to those of K. As is shown in Fig. 1, starting at pH 3.4 and 5.5 for the leaching of bark and leaf biochar, the pH of the leaching solution for these biochars changed to above 7 after a week, to above 8 after 4 weeks. These systematic pH increases, to above 7, could be responsible for the reduced calcium leaching after 1 week: a higher pH hindering the leaching of Ca, including the possible formation of Ca precipitates such as CaCO_3_. For wood biochars in pH 5.5 (initial) solutions, where the pH of leachate solution never reached pH of 7, the concentration of leached Ca does not decrease after 1 week.

The leaching of magnesium tended to be gradual for all 500 °C biochars. Alternatively, the leaching of Mg was more sensitive to pH than that of potassium. It appears that the leaching rate generally decreased with increasing pH value. The leaching in the buffer having an initial pH of 8.5 was the slowest.

#### Aromatic compounds leached out from biochar using buffer solutions

The data presented above on the leaching of biochars with organic solvents reflect broadly the upper limits of organics that might be leached out from the biochars. As can be clear from the preceding discussion, the possible toxicity of the leachates in soil would depend on the rates at which the organics are released from the biochar as well as the consumption rates of these compounds by various chemical and biological processes in the soil. In order to understand the leaching kinetics, i.e. the rates at which organics are released from biochar, the same leachates that were investigated for macronutrients were investigated for organic aromatic compounds.

**Fig. 3:**
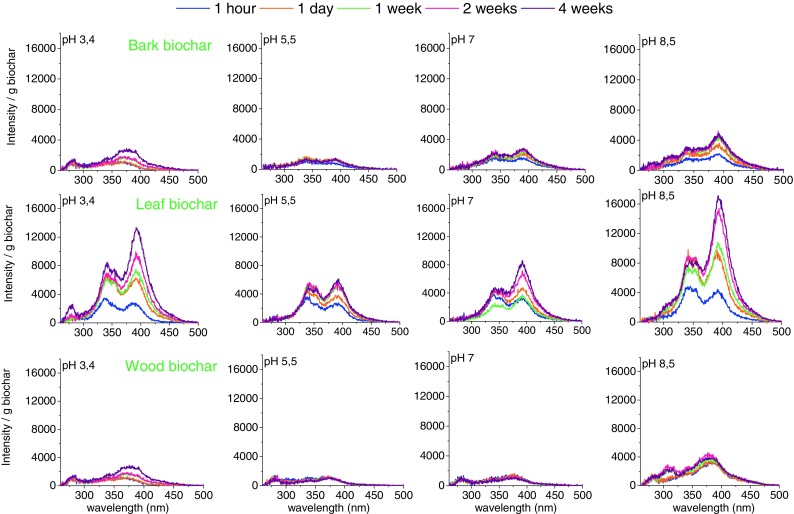
UV fluorescence spectra of bark, leaf and wood (500 °C) biochar of the leaching solutions as a function of leaching time and initial pH value as indicated

Figure 3 shows the UV fluorescence spectra of the leaching solutions as a function of leaching time and leaching solution initial pH value. The fluorescence intensities are expressed on the basis of intensity per gram of biochar. The data are accumulative in nature, e.g. the organics in the leachate in 4 weeks included those of 1 h. To make a better identification of the observed leachate biochar spectra, previous UV-fluorescence studies of coals (Kashimura et al. 2004; Kershaw et al. 2000) and bio-oils (Oudenhoven et al. 2016) were used. Oudenhoven et al. (2016) studied (heavy) pyrolysis oils with UV-fluorescence. To identify the compounds in the spectra, model compounds were investigated and showed that phenolics can be detected between wavelengths of 250–300 nm, naphtol, methoxy naphthalene between 290 and 330 nm, fluoranthene between 350 and 400 nm. The intensity of the observed UV-fluorescence bands is also determined by the polarity of the molecule, e.g. same concentration of naphalene and naphtol, naphtol gives an intensity 5 times higher. Basically, the higher the number of aromatic rings in the compounds, the higher the wavelength at which they give a signal/response.

For all leaching solutions investigated, the leaf biochars gave much higher fluorescence intensity than the wood and bark biochars. The high intensity is either a measure for the concentration and/or polarity of the leached compounds. Hence, leaf biochar when in contact with water like solutions leached out the highest concentration of complex aromatic compounds or the most polar compounds. Further, the UV fluorescence intensity of each leachate seemed to be determined by the pH of the initial solution, as high intensities were found for leachates with an initial pH of 8.5. However, the pH of each of the leaf leachates is above 8 after 4 weeks, seems to indicate that the buffering agents (Table 1) have a bigger effect on the leachability of complex aromatic organic compounds.

The leachate contained relatively large aromatic ring systems, naphthalene-like and fluoranthene-like compounds, respectively, as represented by the fluorescence shoulder and peaks around 340 and 390 nm, respectively. To a lesser extent, mono-aromatic ring structures were found in the leachates, based on the low intensities found around 270 nm. It should be noted that what is observed by UV fluorescence spectroscopy is not necessarily the same molecules as those identified by GC-MS. In particular, the heavier components, likely containing fused aromatic rings, seen with UV-fluorescence spectroscopy have little chance to pass through the GC column to be detected in GC-MS analysis. The data in Fig. 3 clearly show the presence of complex aromatic ring structures in the leachates.

For biochars, more complex aromatic ring structures were leached into the solution at pH of 8.5, as can be seen from the small band around 310 nm. While the exact reason remains unclear, the changes in solubility of many organic molecules/structures with pH must have been at least partly responsible for the observed changes in the fluorescence intensity. In fact, many organic structures such as acidic, phenolic, basic N-containing functionalities have higher solubilities in aqueous solutions at higher pH values. For example, the carboxylic acids, having low solubility in acidic solution, would have increased solubilities at pH as they are (partly) converted into carboxylates. Possibility also exists that some organic (aromatic) structures could have been oxidised and degraded in strong alkali solutions. The leaching of both organic and inorganic species, possibly exceeding the buffering capacity, changed the pH of the leaching solution during the leaching process. In particular, the leaching buffer solution with an initial pH of 3.4 experienced a rapid increase in pH to values close to or even above 8. This explains why the final leachate showed an UV-fluorescence intensity close to that for the initial pH values of 8.5.

The UV-fluorescence data in Fig. 3 are also an indication of the kinetics of the organic matter being leached out of the biochars. The leaching of organics, especially from leaf biochar, was always a slow and gradual process. It appears that the access of water into the micro pores of the biochars as well as the dissolution of organics out of the micro pores in the biochars may have significant activation energy. Alternatively, the leaching of organics involves not only physical process (dissolution and diffusion) but also chemical and/or biochemical reactions (e.g. hydrolysis), which are slow at room temperature. Further research is warranted to understand the nature of the leaching process.

In general, although that bark, leaf and wood biochars are produced under the same conditions, UV-fluorescence profiles for these fluidised-bed biochars show that the concentration, nature and kinetics of the leached aromatic organics from biochars depend on the pH (buffer solution) in which they were leached, and the biochar feedstock.

## Conclusions

A variety of biochars prepared from the pyrolysis of mallee bark, leaf and wood in a fluidised-bed pyrolyser have been leached with aqueous solutions of varying pH values. The leaching time ranged from 1 h to 4 weeks. The initial pH values of the leaching solutions (3.4, 5.5, 7, 8.5) cover the range of the soil pH in the WA Wheatbelt fields where mallee trees are grown and biochars would be returned. The aim of this study was three-fold: firstly to investigate the potential toxicity due to light bio-oil compounds leached from the biochars. Secondly, to examine the leachability of macronutrients and thirdly, complex organic aromatic compounds, when in contact with different pH solutions.

The chemical build-up of bark, leaf and bark biochar is different. Although they are produced under similar conditions, they retain different compounds and different concentrations of bio-oil compounds. Phenolics adhered to biochar surfaces have to be considered when added to soils since the biomass species of which they originate will lead to different concentrations in compounds, e.g. phenolics, which could potentially be leached to the soils and/or surrounding waterways. In this study, wood biochars show the largest potential of leaching phenolics to the soil in which it is placed.

The bark, leaf and wood biochar were placed in different pH solutions showing that the kinetics and concentration of leachable macronutrients and aromatic concentrations will be determined by the biomass feedstock and the buffer capacity of the leaching solution. Biochars liming effect depends largely on the leachability of AAEM to the soil solution; however, there is some evidence of bio-oil compounds, e.g. acetic acid are having an effect on pH of soil solutions. Since the biochars gave rise to the initial pH of the leachates, to pH higher than 8, still the macronutrients and aromatic compounds showed distinct kinetic leaching differences. K and Mg are leached gradually with Mg was more sensitive to pH than that of potassium. The leaching of Ca was very minute likely as a result of Ca being present as CaCO_3_, insoluble at pH > 5.8.

As for the leaching of aromatics together with macronutrients: the leaf biochars gave much higher fluorescence intensity than the wood and bark biochars. The high intensity is either a measure for the concentration and/or polarity of the leached compounds. Further, the UV fluorescence intensity of each leachate seemed to be determined by the pH of the initial solution, as high intensities were found for leachates with an initial pH of 8.5. However, the pH of each of the leaf leachates is above 8 after 4 weeks, seems to indicate that the buffering agents have a bigger effect on the leachability of complex aromatic organic compounds.
